# Specific Foods Associated with Depressive Symptoms among Young Adults and Their Bioactive Effects

**DOI:** 10.3390/nu16121818

**Published:** 2024-06-10

**Authors:** Junghyun Park, Hae-Jeung Lee

**Affiliations:** 1 Institute for Aging and Clinical Nutrition Research, Gachon University, Seongnam 13120, Republic of Korea; iwbstill@yahoo.com; 2 Department of Food and Nutrition, College of BioNano Technology, Gachon University, Seongnam 13120, Republic of Korea; 3 Department of Health Sciences and Technology, Gachon Advanced Institute for Health Sciences and Technology (GAIHST), Gachon University, Incheon 21999, Republic of Korea; 4 Clinical Research Center, Gachon University Gil Medical Center, Incheon 21999, Republic of Korea

**Keywords:** depressive symptoms, specific foods, young adults, bioactive compounds

## Abstract

Depression represents a widespread and devastating psychiatric public health challenge globally. It is particularly prevalent among young adults in Korea. Certain foods may have medicinal properties that alleviate depressive symptoms. This study aimed to examine the association between specific foods and depressive symptoms among young adults, exploring their bioactive effects and possible mechanisms. We conducted a cross-sectional study involving 1000 Korean young adults aged 18–39 years. Food frequency questionnaires were used to assess diets and their associations with depressive symptoms. Results from multivariable logistic regression analysis indicated associations between several specific foods and their effects: milk (odds ratio = 0.58, 95% confidence interval: 0.36–0.94), eggs (0.55, 0.35–0.87), bananas (0.58, 0.36–0.94), oranges (0.62, 0.40–0.96), sweet potatoes (0.60, 0.37–0.97), mushrooms (0.53, 0.31–0.92, females only), and kimchi (0.40, 0.17–0.95, males only). Furthermore, molecular docking indicated that hesperidin had the highest docking score of 5.86 in oranges. Several bioactive compounds identified as potentially beneficial in combatting depression include calcium, casein, alpha-lactalbumin, tryptophan (TRP), vitamin B6 and B12, magnesium, flavonoids (especially hesperidin), carotenoids, ergothioneine, fiber, and probiotics. To recommend these foods in the management of depression among young adults, further clinical intervention studies are necessary.

## 1. Introduction

Depression is a prevalent mental disorder and a significant contributor to the global burden of disease [1]. The number of incident cases of depression worldwide was 258 million in 2017, representing a 49.86% increase from 172 million in 1990 [2]. Moreover, the World Health Organization (WHO) estimates that approximately 280 million people were affected by depression in 2023 [3]. In South Korea, depression ranks as the third leading cause of disability-adjusted life years and is a major risk factor for suicide across all demographics, including young adults. Among the countries of the Organization for Economic Co-operation and Development (OECD), South Korea reports the highest suicide rate [4]. Risk factors for the onset of depression include neurotransmitter depletion, cognitive processes, behavior patterns, disposition, genetic factors, certain personality traits, environmental stressors such as significant life events, and certain sociodemographic factors, notably female gender [5,6].

The most commonly prescribed medicines for depression are serotonin and norepinephrine reuptake inhibitors (SNRIs) and selective serotonin reuptake inhibitors (SSRIs). Traditional Western antidepressants primarily include tricyclic antidepressants (TCAs), monoamine oxidase inhibitors (MAOIs), and norepinephrine reuptake inhibitors (NRIs) [7]. They achieve their antidepressant effects by targeting different neurotransmitter systems, such as serotonergic (5-HT), dopaminergic (DA), and noradrenergic (NA), or by inhibiting monoamine oxidase (MAO) enzymes [8]. However, these antidepressants may commonly lead to a range of side effects such as insomnia, headaches, excessive sweating, dry mouth, nausea, constipation, diarrhea, loss of appetite, weight loss, dizziness, and sexual dysfunction. In the case of SSRIs, fatigue, drowsiness, weight gain, and increased anxiety or agitation (especially at the beginning of treatment) were additionally reported. For TCAs, blurred vision, urinary retention, drowsiness, weight gain, low blood pressure when standing, and increased heart rate were also reported. For MAOIs, drowsiness, lightheadedness, weight gain, and hypertensive crisis when taken with certain foods or medications were additionally reported [6,9]. Therefore, due to these side effects, many patients with depression fail to complete their medication regimens despite needing treatment. This leads to frequent disruptions in their therapy [10].

It is widely recognized that foods can influence brain functions, including cognition and emotional processing. Although no single food or specific diet can entirely replace antidepressants [11], the potential of non-pharmacological foods to alleviate depressive symptoms should not be overlooked. Such foods are typically well-tolerated, with minimal side effects, and are both readily available and affordable. The beneficial effects of various micronutrients, including B-complex vitamins, vitamin D, folate, and omega-3 3-fatty acids, on depressive and mood disorders have been well-documented [12,13,14]. The mechanisms underlying these associations generally involve interactions with stress hormones, stress-related pro-inflammatory cytokines, and neurotransmitters [15]. However, as the field of nutritional psychiatry and the study of the ‘microbiome–gut–brain axis’ are relatively new [16], the impacts of specific foods, nutrients, and bioactive components, including phytochemicals and psychobiotics, warrant further investigation through diverse study designs across different age groups.

Throughout the entire life cycle, strong epidemiological evidence indicates that poor diet or unhealthy foods are associated with depression, especially in young adults who are more susceptible to poor diet compared to other age groups [17,18]. Among balanced diets or healthy foods for young adults, certain foods with bioactive components have the potential to be candidate components of safe antidepressant agents. This study originated from the idea of identifying specific foods with effective bioactive components that target young adults with elevated depressive symptoms. Specifically, this study aimed to explore the association between certain single foods and depressive symptoms among young adults in South Korea via a cross-sectional survey.

## 2. Materials and Methods

### 2.1. Study Design and Participants

In this cross-sectional study, 1000 Korean young adults aged 18–39 years residing in Seoul and Gyeonggi-do were enrolled. Recruitment occurred through community colleges and universities via visits, as well as through flyers, poster advertisements, and postings on student bulletin boards and websites. The enrollment and data collection phases spanned from March to June 2017, commencing only after obtaining informed consent from the participants. The Center for Epidemiologic Studies Depression Scale (CES-D), a well-established psychiatric questionnaire, was used to identify participants as either normal or exhibiting depressive symptoms, with a cutoff point set at 16 [19]. Exclusions were made for participants who either failed to complete the CES-D (*n* = 2) or reported extreme caloric intake (≤800 or ≥4000 kcal/day for males and ≤500 or ≥3500 kcal/day for females; *n* = 142) [20]. The final sample comprised 856 individuals, divided into two groups based on the CES-D threshold: the normal group (*n* = 541) and the depression group (*n* = 315).

### 2.2. Data Collection

We designed a comprehensive questionnaire encompassing demographic data, mental health status, a Food Frequency Questionnaire (FFQ), and anthropometric measures. Demographic data included gender, age, type of residence, and income levels. Mental health status was assessed through participant responses concerning stress level, feelings of sadness or despair, experiences with psychological counseling, suicidal ideation, plans for suicide, and suicide attempts. Types of residence were classified as living alone, with a roommate, spouse, parents, grandparents, or other arrangements. Household income was categorized into five groups: very low (less than 1 million won per month), low (1 million to less than 3 million won per month), medium (3 million to less than 4 million won per month), medium-high (4 million to less than 6 million won per month), and high (over 6 million won per month). Current stress levels were categorized as ‘too much high’, ‘pretty high’, ‘little’, and ‘almost no stress’. Questions regarding ‘sadness or despair feelings’, ‘counseling experience’, ‘suicidal ideation’, ‘suicide planning’, and ‘suicide attempts’ were posed in a dichotomous ‘yes’ or ‘no’ format. Anthropometric data, including height, weight, body mass index (BMI), body fluid, protein, mineral, and body fat percentage, were collected using the InBody 720 device (Biospace Co., Ltd., Seoul, Republic of Korea). Additionally, arterial blood pressure was measured in a seated position after 5 min of rest.

### 2.3. Dietary Assessment

The dietary assessment to examine foods related to depressive symptoms was carried out using a 112-item FFQ. The FFQ consists of 112 common foods and dish items. Participants responded to categories indicating the frequency of consumption during the previous year, which were divided into nine levels: none, 1 time/month, 2–3 times/month, 1 time/week, 2–4 times/week, 5–6 times/week, 1 time/day, 2 times/day and ≥3 times/day. Following that, participants were asked to select one of four average portion sizes: small (0.5), medium (1.0), medium-large (1.5), and large (2.0). This questionnaire has been validated and used for the Korea National Health and Nutrition Examination Survey (KNHANES) [20].

### 2.4. Depressive Symptoms

Depressive symptoms were assessed using the CES-D scale. The scale is designed with 20 items and is a self-report scale used to measure depressive symptomatology experienced during the previous week [15]. The CES-D is known to be a valid and reliable assessment tool for the general population experiencing clinically relevant depression (CES-D score ≥ 16) [19,21].

### 2.5. Statistical Analysis

The general characteristics of the participants were presented as percentages or means ± standard deviations. Statistical analyses of categorical variables were conducted using the chi-squared test, whereas continuous variables were analyzed using the Student *t*-test. Fisher’s exact test was utilized instead of the chi-squared test when more than 20% of cells had expected frequencies below 5. Multivariable logistic regression was employed to examine the associations between individual food intake and depressive symptoms, adjusting for age, sex, and income in the regression models. All statistical analyses were performed using SAS software (SAS version 9.3, SAS Institute, Cary, NC, USA).

### 2.6. In Silico Prediction Molecular Docking

We used in silico prediction molecular docking to identify bioactive substances in specific foods based on results from the LucyNet GAIA database, which consists of a 400-billion chemical library and target information from PharminoGen Inc., Yongin-si, Republic of Korea. First, among the specific foods found to be associated with depressive symptoms in young Korean adults, in silico screening was performed for components that might have antidepressant effects. The next step was to narrow down the substances targeting BDNF agonists and MAO inhibitors. This process was conducted using LucyNet HUB, a large-scale database that includes interactions between compounds, omics information, and target proteins based on deep learning. The Hub module consists of LucyNet Kinase, LucyNet Onco, LucyNet GPCR, and LucyNet PLX. By entering compound information through the web UI, predictive values for cells and target proteins were obtained. Flavonoid compounds, among several compounds from these foods, were analyzed for molecular modeling of phytochemicals targeting MAOIs (Monoamine Oxidase Inhibitors). The entire molecular docking process was performed by PharminoGen Inc., Yongin-si, Republic of Korea.

## 3. Results

### 3.1. General Characteristics of Participants

Cross-tabulation analysis revealed significant demographic and psychosocial differences between the groups. The proportion of women in the depressed group was significantly higher (69.5%) compared to the normal group (59.6%). Additionally, the proportions of ‘very low’ and ‘low’ household income were more prevalent in the depressed group. Emotional distress indicators such as ‘sad and desperate feelings’ were notably higher in the depressed group (28.6%), as were stress levels, with ‘too much high’ stress reported at 8.3% versus 0.4% and ‘pretty high’ stress at 47.6% versus 20.0% in the normal group. Rates of serious ‘suicidal ideation’ and ‘suicide planning’ were also elevated in the depressed group. Interestingly, ‘counseling experience for psychological problems’ was more frequently reported in the normal group. Anthropometric measurements showed that the depressed group had significantly lower height, weight, body water, body protein, and body minerals compared to their counterparts. However, no significant differences were observed in BMI, body fat, or blood pressure between the two groups (Table 1).

### 3.2. Association between Food Intake and Depression

Following the conversion of food intake frequencies into weekly units using a 112-item Food Frequency Questionnaire (FFQ), the data were stratified into quintiles using the ‘PROC RANK’ procedure in SAS (Statistical Analysis System). Food intake frequencies were categorized into four or five groups based on the distribution of individual food intake frequencies. After assessing model suitability and adjusting for potential confounders, multivariable logistic regression analysis revealed significant associations between high intake of certain foods and lower odds of depressive symptoms. Specifically, the highest intake quintiles of ‘milk’ (Odds Ratio = 0.58, 95% Confidence Interval: 0.36–0.94), ‘eggs’ (OR = 0.55, 95% CI: 0.35–0.87), ‘bananas’ (second highest intake, OR = 0.58, 95% CI: 0.36–0.94), ‘oranges’ (OR = 0.62, 95% CI: 0.40–0.96), and ‘sweet potatoes’ (OR = 0.60, 95% CI: 0.37–0.97) were inversely associated with depressive symptoms. Among females, the highest intakes of ‘mushrooms’ (OR = 0.53, 95% CI:0.31–0.92), ‘eggs’ (OR = 0.45, 95% CI:0.26–0.80), and ‘milk’ (OR = 0.57, 95% CI:0.34–0.95) also indicated a lower prevalence of depressive symptoms. In males, the second-highest intake of kimchi was inversely associated with depressive symptoms. Notably, the associations for bananas and kimchi did not demonstrate significant *p* for trends. The results of these specific foods showing significant associations with depressive symptoms are detailed in Table 2.

### 3.3. In Silico Prediction of Antidepressant Phytochemicals

**Figure 1:**
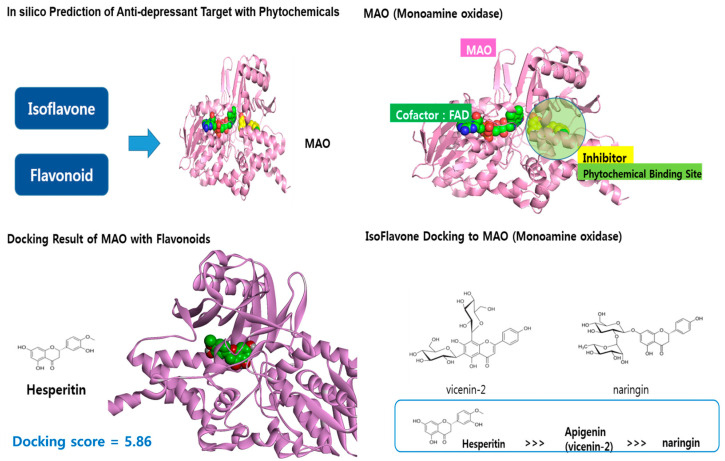
In silico prediction of anti-depressant target with phytochemicals.

After in silico screening using a chemical components database, substances targeting BDNF agonists and MAO inhibitors were identified among the specific foods associated with depressive symptoms in young Korean adults. Among the flavonoids contained in oranges, hesperidin showed relatively superior inhibitory activity against MAO, presenting a docking score of 5.86 (Figure 1), compared to the docking scores of 4.31–4.97 for carotenoids docking to BDNF. Vicenin 2 and naringin also exhibited mild MAO inhibitory activity, suggesting they may have antidepressant effects.

## 4. Discussion

Based on the results of this study, when elucidating the mechanisms of specific single foods related to depressive symptoms, it was found that milk contains bioactive components such as tryptophan (TRP), casein, calcium, α-lactalbumin (α-La), vitamin B6 and B12, and folate. These components derived from milk have been reported to affect depression. A double-blind cross-over clinical trial reported that a TRP-rich hydrolyzed protein significantly improved mood and attenuated the cortisol response to acute stress. This may occur by augmenting brain TRP and promoting stress resilience [22]. Joung et al. indicated that treatment with milk casein improves unpredictable chronic mild stress-induced impairment of neuroendocrine function by regulating the hypothalamic-pituitary-adrenal (HPA) axis [23]. A young female with premenstrual syndrome (PMS) randomized controlled trial (RCT) explained that calcium supplements can restore calcium homeostasis and, in turn, prevent depression based on intracellular calcium concentration and reduce mood disorders during PMS [24]. A cross-over RCT demonstrated that consuming a drink containing α-La and carbohydrates led to an increase in the plasma TRP-large neutral amino acids (LNAA) ratio, which can result in elevated TRP uptake in the brain, leading to higher serotonin levels [25]. Milk and eggs are rich in vitamins B6 and B12. A cross-sectional study highlighted the association between deficient vitamin B6 intake and depression among older women (OR = 6.19, 95% CI: 1.17–32.76) [26]. Another cross-sectional study reported an inverse association between vitamin B6 and depressive symptoms in both boys (OR = 0.73, 95% CI: 0.54–0.98, *p* for trend = 0.02) and girls (OR = 0.72, 95% CI: 0.56–0.92, *p* for trend = 0.002) [27]. Both studies suggested plausible mechanisms by which vitamin B6 affects the metabolism of homocysteine and the synthesis of monoamine neurotransmitters such as dopamine, norepinephrine (NE), and serotonin in the brain. As for eggs, accumulated evidence has shown that the predominant bioactive component influencing depressive symptoms is also TRP. TRP-rich egg protein plays a role in increasing the plasma TRP–LNAA ratio [28]. The increased TRP-LNAA ratio increases brain serotonin levels and functional serotonin activity, which can also lead to higher brain noradrenaline levels and improved mood [29]. A previous cross-sectional study using SUN (The Seguimiento Universidad de Navarra/University of Navarra Follow-up) cohort data suggested an inverse association between vitamin B12 intake and the prevalence of depression. Possible mechanisms include the involvement of vitamin B12 in depression associated with neurochemical pathways and single-carbon transfer reactions required for the production of serotonin, monoamine neurotransmitters, and catecholamines [30].

Bananas are relatively inexpensive and readily available; they are also rich in neurotransmitters such as N-acetyl serotonin, dopamine, catecholamine, and the precursor amino acid, including TRP [31,32]. Notably, the phyto-antioxidant present in bananas has an impact on reducing anxiety-like conditions and normalizing the HPA axis [32]. Bananas are a good source of several minerals with known antidepressant properties, including magnesium [33], iron [34], and potassium [35]. A Japanese cross-sectional study reported an inverse association between the medium category of banana intake and depression (OR = 0.62, 95% CI: 0.41–0.95) and explained that a magnesium deficiency can result in dysregulation of the HPA axis and anxiety [36]. A prospective longitudinal study demonstrated that the participants in the middle tertile of dietary magnesium intake (adjusted mean: 414.3 ± 16.2 mg/d) had a statistically significant lower risk of receiving a hospitalization diagnosis of depression (adjusted hazard ratio = 0.49, 95% CI: 0.25–0.95, *p* = 0.035). The study indicated that magnesium is involved in the regulation of the HPA system, believed to be the main stress response system.

Orange juice contains potent antioxidants, including flavonoids (hesperidin, naringenin), carotenoids (xanthophylls, cryptoxanthins, carotenes), and vitamin C [37]. Fresh orange juice contains approximately 30 mg of hesperidin per 100 mL [38]. Similar to the results from molecular docking, a previous preclinical study reported that hesperidin treatment inhibited MAO activity and decreased TRP hydroxylase-1 expression in the hippocampus [39]. A previous RCT with a flavonoid-rich orange juice intervention concluded that alterations in the gut microbiome due to flavonoid treatment might be responsible for a potential improvement in depression among young adults. Additionally, the study showed that the abundance of *Lachnospiraceae* increased after intervention, emphasizing the ability of *Lachnospiraceae* to break down carbohydrates into short-chain fatty acids, which regulate neurotransmitter levels. This process ultimately led to enhanced neuroprotection and improved penetration of the blood–brain barrier (BBB) [40]. Beta-carotene is a strongly colored red-orange pigment and a source of vitamin A with antioxidant and free radical scavenging potential. The color of sweet potato flesh varies; it can be white, yellow, purple, or orange. Among these, the orange-fleshed sweet potato (*Ipomoea batatas* L.) is particularly high in carotenoids. It has been reported that orange-fleshed sweet potato is a second staple crop in several African and Asian countries. Specifically, α and ß-carotenes are found at very high levels in orange-fleshed sweet potatoes compared with other commonly consumed vegetables and fruits. Preclinical research with ß-carotenes has shown the potential of ß-carotene as a novel therapeutic agent for depression. In the group administered ß-carotene, levels of brain-derived neurotrophic factors (BDNF) and NE were significantly increased [41]. Given that BDNF plays a role in enhancing neurogenesis by up-regulating synaptic functions and neuron cell survival in nervous systems, depletion of BDNF leads to the pathology and physiology of depression. NE is responsible for regulating emotions, and a deficiency of NE in the brain contributes to the recurrence of depressive symptoms after treatment with NE-based antidepressants [42].

Mushrooms are low in calories and contain numerous bioactive compounds, including antioxidants, anti-inflammatory agents, vitamin B complex, and vitamin C. Among these, ergothioneine (ERGO) is a hydrophilic antioxidant found at high levels in mushrooms. Depressed patients often exhibit increased oxidative stress and lower levels of antioxidants. Data from NHANES 2005–2016 demonstrated that individuals in the middle tertile of mushroom intake had lower odds of depression (adjusted OR = 0.31, 95% CI 0.16–0.60) compared with those in the lowest tertile of intake [43]. Thus, ERGO may play a significant role in the gut microbiome–brain axis in preventing depression. Kimchi is a popular fermented food rich in probiotics such as *Lactobacillus*, *Leuconostoc*, and *Weissella*. KNHANES data 2012–2016 showed that the highest tertile of probiotic food consumption, including kimchi, had significantly lower odds with respect to depression severity (adjusted OR = 0.48, 95% CI: 0.28–0.81) [44]. Accumulated evidence suggests that gut microbiota modulate systemic inflammation [45] and play a crucial role in regulating mood activity through various mechanisms [46]. Bacteria that produce neurotransmitters can significantly influence neural biochemistry, mood, and behavior [44]. 

These bioactive compounds derived from the specific foods can exhibit synergistic effects with each other. (1) Vitamin B6 and B12: As we mentioned above, these two vitamins play an important role in the synthesis of neurotransmitters. They are essential for the production of mood-regulating neurotransmitters such as serotonin and dopamine. When vitamin B6 and B 12 work together, they support neural health and can help alleviate symptoms of depression [47]. (2) Magnesium and TRP: When taken together with magnesium, serotonin synthesis is enhanced, which can be effective in improving depression [48]. (3) Flavonoids (especially hesperidin) and vitamin C: Hesperidin and vitamin C, found in fruits like oranges, possess strong antioxidant effects and can help reduce inflammation in the brain. When these two components work together, they can enhance neuroprotective effects and positively impact the management of depression [49]. (4) Probiotics and fiber: These play a critical role in improving gut-brain axis health and regulating immune responses. When fiber-rich foods and probiotics are consumed together, they promote the growth of beneficial bacteria in the gut, which can help alleviate symptoms of depression [50]. Therefore, when composing meals or developing health supplements and antidepressants, considering these synergistic effects could yield more positive results in the prevention of depression. 

**Figure 2:**
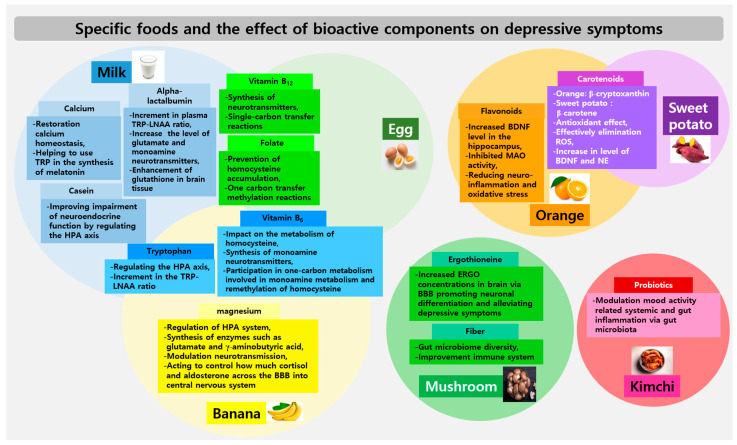
Specific foods and the effect of bioactive components on depressive symptoms. The intersected areas within each food’s circles indicate cases where the same components are shared. BBB: blood–brain barrier, BDNF: brain-derived neurotrophic factors, ERGO: ergothioneine, HPA: hypothalamic–pituitary–adrenal, LNAA: large neutral amino acids, NE: norepinephrine, ROS: reactive oxygen species, TRP: tryptophan.

Depression has been reported to be associated with oxidative stress [51], inflammation [52], high levels of homocysteine [53], deficiencies in serotonin or catecholamine [54,55], impaired neurodevelopment [56], and an abnormal HPA axis [57]. Most bioactive components derived from specific foods are related to the above-mentioned mechanisms (Figure 2). The specific foods that are beneficial for depression were selected from the results of numerous cross-sectional studies, often including the same bioactive compounds. Foods containing bioactive compounds that affect depression are commonly used in daily meals and are easily accessible. One study reported that the diet quality of Korean young adults was low [58]. The importance of a balanced diet that includes diverse foods cannot be overlooked; indeed, dietary quality is well-known to play a pivotal role in preventing depression. A previous study asserted that better diet quality is associated with a lower occurrence of major depressive disorder (MDD), particularly among young adults [59]. To prevent and manage depressive symptoms, it is helpful to initially focus on achieving a balanced diet and consuming specific foods rich in bioactive compounds that affect depressive symptoms, as found in this study. However, the causality between a balanced diet and depression requires further study. Depression-related poor appetite or the regulation of emotions through the consumption of unhealthy foods, including sweets and fast foods, can limit the ability of individuals with depression to achieve a more balanced diet [60,61]. Moreover, since most bioactive compounds occur in small quantities in specific foods, the amounts are likely too small to actively suppress depression symptoms within a balanced diet alone. It is possible to extract these bioactive compounds and use them to develop functional foods or effective antidepressant medications with fewer side effects by blending single or multiple compounds, especially for young adults. However, much more research on this topic is required.

This is the first study to analyze specific foods related to depressive symptoms in Korean young adults and to elucidate the effects and plausible mechanisms of bioactive compounds from specific foods: milk, egg, banana, orange (hesperidin), sweet potato, mushroom, and kimchi. However, there are several limitations to this study. Due to its cross-sectional design, identifying causal relationships between specific foods and depressive symptoms requires further clarification. Moreover, given the nature of this study and the absence of depressive patients as study participants, it was challenging to establish clear results regarding the cited associations. Further research is required to explore the causal relationships and underlying mechanisms of bioactive components in specific foods concerning the prevention of depressive symptoms. Individual bioactive components, along with various combinations of these components, should be meticulously investigated to develop potential antidepressants or functional foods for young adults with depression.

## 5. Conclusions

Milk, eggs, bananas, oranges, sweet potatoes, mushrooms, and kimchi showed beneficial associations with depressive symptoms in young Korean adults via a cross-sectional study, although bananas and kimchi did not show significant *p* for trends. Calcium, casein, and alpha-lactalbumin in milk; TRP, vitamin B6, and B12 in milk, eggs, and bananas; magnesium in bananas; flavonoids (especially hesperidin) in oranges; carotenoids in oranges and sweet potatoes; ERGO and fiber in mushrooms; and probiotics in kimchi have been identified as bioactive compounds that could potentially be beneficial for depression. Possible mechanisms have been suggested for the involvement of these bioactive components. In light of these findings, further well-designed clinical intervention studies are required to verify the efficacy of these bioactive compounds in treating depression.

## Figures and Tables

**Table 1:** The characteristics of participants.

Variables		Normal (N = 541)	Depressive Symptoms (N = 315)	*p*-Value
Percentage (%)				
Women		59.6	69.5	0.004
Age	18–21 years	51.8	43.8	0.072
	22–29 years	47.7	55.2	
	30–39 years	0.6	1.0	
Family type	Living alone	10.8	10.2	0.218
	With roommate	15.0	12.1	
	With spouse	0.0	0.6	
	With parents or grandparents	71.4	75.2	
	Other	2.8	1.9	
Income (household)	Very low	4.7	5.1	0.172
	Low	16.2	22.5	
	Medium	18.4	17.8	
	Medium-high	37.7	35.9	
	High	23.1	18.7	
Stress levels	Too much high	0.4	8.3	<0.001
	Pretty high	20.0	47.6	
	Little	65.9	42.2	
	Almost no stress	13.7	1.9	
Feelings of sadness or despair	Yes	5.0	28.6	<0.001
	No	95.0	71.4	
Experiences with psychological counseling	Yes	18.5	12.4	0.524
	No	81.5	87.6	
Suicidal Ideation	Yes	0.6	10.2	<0.001
	No	99.4	89.8	
Plans for suicide	Yes	0.0	1.9	0.002
	No	100.0	98.1	
Suicide attempts	Yes	0.0	0.6	0.136
	No	100.0	99.4	
Mean ± S.D.				
Height		166.1 ± 8.2	164.5 ± 8.3	0.010
weight		61.3 ± 11.5	59.5 ± 11.8	0.025
BMI (kg/m^2^)		22.1 ± 2.8	21.8 ± 2.9	0.176
Body fluid		33.5 ± 7.6	31.9 ± 7.4	0.002
Protein		9.0 ± 2.1	8.6 ± 2.0	0.002
Mineral		3.3 ± 0.7	3.1 ± 0.7	0.012
Body fat		15.5 ± 5.3	15.9 ± 5.6	0.279
SBP		121.6 ± 12.2	120.2 ± 12.0	0.121
DBP		71.2 ± 9.3	71.4 ± 9.6	0.789

The chi-square test or Fisher’s exact test was applied for categorical variables, and the Student’s *t*-test was applied for continuous variables.

**Table 2:** Association between the selected food intake frequency and depressive symptoms.

Food	Total											Men											Women										
	Group	N	Median (per Week)	Crude			*p* for Trend	Multivariable Adjusted			*p* for Trend	Group	N	Median (per Week)	Crude			*p* for Trend	Multivariable Adjusted			*p* for Trend	Group	N	Median (per Week)	Crude			*p* for Trend	Multivariable Adjusted			*p* for Trend
				OR	Lower CL	Upper CL		OR	Lower CL	Upper CL					OR	Lower CL	Upper CL		OR	Lower CL	Upper CL					OR	Lower CL	Upper CL		OR	Lower CL	Upper CL	
Milk	0	165	0.00		REF		0.023		REF		0.038	0	49	0.00		REF		0.059		REF		0.071	0	116	0.00		REF		0.106		REF		0.90
	1	115	0.58	0.72	0.44	1.17		0.73	0.45	1.19		1	74	1.00	0.86	0.41	1.81		0.86	0.40	1.87		1	83	0.58	0.78	0.44	1.38		0.80	0.46	1.42	
	2	140	1.00	0.68	0.43	1.08		0.72	0.45	1.15		2	98	3.00	0.63	0.31	1.30		0.66	0.31	1.38		2	98	1.00	0.54	0.31	0.94		0.56	0.32	0.98	
	3	298	3.00	0.57	0.39	0.84		0.61	0.41	0.90		3	35	5.50	0.55	0.21	1.42		0.57	0.21	1.49		3	114	3.00	0.67	0.40	1.14		0.67	0.40	1.13	
	4	138	7.00	0.56	0.35	0.90		0.58	0.36	0.94		4	58	7.00	0.50	0.22	1.15		0.51	0.22	1.20		4	131	7.00	0.57	0.34	0.95		0.57	0.34	0.95	
Eggs	0	181	0.00		REF		0.047		REF		0.020	0	76	0.00		REF		0.983		REF		0.739	0	105	0.00		REF		0.010		REF		0.009
	1	160	0.23	0.76	0.49	1.18		0.77	0.49	1.20		1	66	0.23	0.79	0.39	1.60		0.85	0.41	1.75		1	94	0.23	0.74	0.42	1.30		0.74	0.42	1.30	
	2	206	0.58	0.80	0.53	1.21		0.79	0.52	1.20		2	64	0.58	0.66	0.32	1.36		0.69	0.33	1.47		2	142	0.58	0.81	0.48	1.34		0.82	0.49	1.38	
	3	144	1.00	1.00	0.64	1.56		0.99	0.63	1.55		3	53	1.00	0.65	0.30	1.41		0.69	0.31	1.51		3	91	1.00	1.19	0.68	2.10		1.19	0.68	2.09	
	4	165	3.00	0.59	0.38	0.92		0.55	0.35	0.87		4	55	3.00	0.88	0.42	1.84		0.82	0.38	1.74		4	110	3.00	0.46	0.26	0.81		0.45	0.26	0.80	
Bananas	0	140	0.00		REF		0.303		REF		0.225	0	62	0.00		REF		0.309		REF		0.203	0	124	0.02		REF		0.524		REF		0.516
	1	282	0.58	0.80	0.53	1.21		0.72	0.47	1.09		1	48	0.23	0.72	0.33	1.60		0.71	0.31	1.60		1	142	0.58	0.75	0.46	1.22		0.73	0.45	1.20	
	2	144	1.00	0.90	0.56	1.44		0.85	0.53	1.38		2	92	0.79	0.66	0.33	1.30		0.63	0.31	1.27		2	98	1.00	0.88	0.52	1.51		0.92	0.53	1.57	
	3	165	3.00	0.61	0.38	0.98		0.58	0.36	0.94		3	68	3.00	0.57	0.27	1.20		0.57	0.27	1.23		3	97	3.00	0.61	0.35	1.05		0.60	0.35	1.05	
	4	125	7.00	0.77	0.47	1.27		0.69	0.42	1.15		4	44	7.00	0.59	0.26	1.37		0.51	0.21	1.20		4	81	7.00	0.81	0.46	1.43		0.81	0.46	1.43	
Oranges	0	252	0.00		REF		0.086		REF		0.045	0	97	0.00		REF		0.272		REF		0.285	0	155	0.00		REF		0.052		REF		0.055
	1	117	0.23	0.67	0.43	1.07		0.66	0.41	1.06		1	18	0.05	0.68	0.22	2.07		0.63	0.20	2.05		1	73	0.23	0.85	0.48	1.49		0.85	0.49	1.50	
	2	104	0.23	1.15	0.72	1.83		1.15	0.71	1.83		2	69	0.23	0.67	0.34	1.32		0.65	0.33	1.30		2	115	0.23	1.11	0.68	1.81		1.08	0.66	1.76	
	3	237	0.68	0.70	0.48	1.01		0.72	0.49	1.05		3	64	0.58	0.81	0.41	1.58		0.92	0.46	1.84		3	93	0.68	0.55	0.32	0.95		0.58	0.33	1.00	
	4	146	3.00	0.67	0.43	1.02		0.62	0.40	0.96		4	66	1.41	0.62	0.31	1.23		0.60	0.30	1.21		4	106	3.00	0.62	0.37	1.04		0.62	0.37	1.04	
Sweet potatoes	1	364	0.00		REF		0.142		REF		0.047	1	163	0.00		REF		0.429		REF		0.244	1	201	0.00		REF		0.070		REF		0.079
	2	241	0.23	0.90	0.64	1.26																	2	151	0.23	0.82	0.53	1.25		0.84	0.54	1.29	
	3	148	0.58	0.84	0.56	1.25		0.78	0.52	1.17		3	90	0.23	0.96	0.55	1.68		0.82	0.46	1.46		3	114	0.58	0.73	0.46	1.17		0.76	0.47	1.22	
	4	103	3.00	0.70	0.44	1.11		0.60	0.37	0.97		4	61	0.58	0.76	0.39	1.47		0.68	0.34	1.33		4	76	2.00	0.59	0.34	1.03		0.60	0.34	1.05	
Mushrooms	1	386	0.00		REF		0.287		REF		0.108	1	147	0.00		REF		0.307		REF		0.693	1	239	0.00		REF		0.033		REF		0.023
	2	183	0.23	0.77	0.53	1.12		0.71	0.49	1.04		2	69	0.23	1.13	0.60	2.12		0.95	0.50	1.82		2	114	0.23	0.63	0.40	1.00		0.60	0.38	0.96	
	3	162	0.58	0.90	0.62	1.32		0.86	0.59	1.27		3	51	0.58	1.41	0.72	2.78		1.35	0.67	2.70		3	111	0.58	0.70	0.44	1.10		0.70	0.44	1.11	
	4	125	1.00	0.78	0.51	1.19		0.67	0.43	1.04		4	47	1.00	1.33	0.66	2.70		1.03	0.49	2.15		4	78	1.00	0.57	0.34	0.98		0.53	0.31	0.92	
Kimchi	0	127	0.23		REF		0.838		REF		0.843	0	53	0.00		REF		0.350		REF		0.290	0	74	0.23		REF		0.331		REF		0.348
	1	322	3.00	1.07	0.70	1.63		1.00	0.65	1.54		1	93	3.00	0.75	0.37	1.52		0.65	0.31	1.35		1	81	1.00	1.27	0.66	2.43		1.27	0.66	2.43	
												2	57	5.50	0.83	0.38	1.80		0.67	0.30	1.53		2	148	3.00	1.24	0.69	2.21		1.28	0.71	2.29	
	3	278	7.00	0.96	0.62	1.49		0.93	0.60	1.46		3	61	7.00	0.45	0.20	1.02		0.40	0.17	0.95		3	160	6.25	1.31	0.74	2.32		1.31	0.74	2.32	
	4	129	14.00	1.11	0.67	1.85		1.07	0.64	1.80		4	50	14.00	0.71	0.31	1.61		0.63	0.27	1.46		4	79	14.00	1.47	0.77	2.82		1.47	0.77	2.84	

Food intake frequency is calculated per week. Multivariable logistic regression was applied, and age, sex, and income were adjusted for multi-variable logistic regression models.

## Data Availability

All datasets used and/or analyzed during the current study are available from the corresponding author upon reasonable request.
